# Robust high-sensitivity real-time impedance analysis for neutralization drug screening and toxicity kinetics of *Clostridioides difficile* toxin TcdA

**DOI:** 10.3389/fmicb.2026.1939062

**Published:** 2026-09-09

**Authors:** Marta Braghetto, Sarah Line Skovbakke, Lasse Ebdrup Pedersen, Steffen Goletz

**Affiliations:** Biotherapeutic Glycoengineering and Immunology, Section for Medical Biotechnology, Department of Biotechnology and Biomedicine, Technical University of Denmark, Kgs Lyngby, Denmark

**Keywords:** cell-based impedance with real-time cell analysis (CBI-RTCA), *Clostridioides difficile* (*C. difficile*), cytotoxicity, neutralization, toxin A (TcdA), toxin quantification, visual inspection of cell morphology

## Abstract

*Clostridioides difficile (C. difficile*) is the main cause of nosocomial infections. It is responsible for mild to life-threatening severe gastrointestinal infections whose typical symptoms are fluid loss caused by Toxin A (TcdA) and Toxin B. In cell cultures, the two exotoxins provoke cell rounding, a morphological change that is visible under the microscope. This characteristic has been widely exploited in *in vitro* assays to evaluate their cytotoxic effect as well as the neutralizing activity of anti-toxin compounds. However, the evaluation of morphological changes relies on subjective scoring, introducing a risk for bias, limiting reproducibility and sensitivity. In the present study, we demonstrate, optimize and validate the applicability of cell-based impedance with real-time cell analysis (CBI-RTCA) for quantifying the effects of TcdA on Vero cells. CBI-RTCA can be used to monitor changes in cell adhesion and proliferation, which are measured as Cell Index (CI). We assessed the effect of TcdA, and a TcdA-neutralizing antibody, using CBI-RTCA and compared the results with visual scoring readouts from multiple operators. The addition of TcdA to Vero cells caused a dose-dependent decrease in CI with a TcdA detection limit of 0.15 pM. CBI-RTCA was more sensitive than visual morphology assessment, detecting reduced cell adhesion within 1 h of TcdA treatment, compared with 5 h by microscopy. TcdA was neutralized by AH3_Fc antibody and using CBI-RTCA which enabled kinetic measurements of the neutralizing drug. The kinetic readouts reported significantly lower variability than morphological inspection, thereby showing higher reproducibility. In addition, CBI-RTCA was utilized for TcdA quantification. The generation of a dose-response curve which correlated the percentage of cell viability to TcdA concentrations allowed the quantification of the low TcdA concentration (12.5 pM) giving 10% of cell viability. The study demonstrates that CBI-RTCA enables continuous, kinetic measurement of toxic and drug-neutralizing effects throughout the experiment with minimal operational effort, eliminating labor-intensive image sampling and readout bias. Moreover, CBI-RTCA is well suited for the detection and quantification of TcdA, even at low concentrations, making it ideal for drug screening and high-through-put applications and has the potential to be adapted and validated for further toxins.

## 1 Introduction

*Clostridioides difficile (C. difficile*), formerly known as *Clostridium difficile*, is a spore-forming pathogen (Aktories et al., 2017; Cammarota et al., 2019; Raeisi et al., 2023), transmitted via the fecal-oral route in spore form (Czepiel et al., 2019; Schäffler and Breitrück, 2018). *C. difficile* causes infections in the gastrointestinal (GI) tract, specifically in the colon (Aktories et al., 2017; Engevik et al., 2015; Schäffler and Breitrück, 2018). Once *C. difficile* adheres to the GI epithelium, it releases Toxin A (TcdA) and Toxin B (TcdB) (Aktories et al., 2017; Yang et al., 2014), the two main pathogenic factors responsible for clinical symptoms (Aktories et al., 2017; Qiu et al., 2016; Schmidt et al., 2016; Xing et al., 2017). TcdA and TcdB bind epithelial host-cell receptors leading to their internalization via endocytosis (Di Bella et al., 2016; Kordus et al., 2022). Due to a decrease in the endosomal pH, the toxins are subjected to a conformational change which induces their autoproteolysis and glucosylation of Rho proteins (Aktories et al., 2017; Andersen et al., 2015; Di Bella et al., 2016; Kordus et al., 2022). The intracellular signaling ends with the loss of cell cytoskeletal structure which provokes fluid loss (Barthold et al., 2022; Davies et al., 2013; Ok et al., 2023; Valdés et al., 2015).

The ability of TcdA and TcdB to decrease cell viability can be studied *in vitro* using cytotoxicity assays (Chen et al., 2020; Davies et al., 2013; Nguyen et al., 2020; Yang et al., 2014). The cell viability can be evaluated, for instance, by sulforhodamine B (Hernandez et al., 2015; Vichai and Kirtikara, 2006) and WST-1 (tetrazolium salt) assays (Hussack et al., 2023; Stoddart, 2011; Sulea et al., 2018). Visual scoring of the cell morphology is used as the standard procedure (Bernardo et al., 2021; Chen et al., 2020; Gray et al., 2021; Hussack et al., 2011; Yang et al., 2014)and is based on microscopy readouts comparing images of treated cells to cells in healthy conditions. A score is then assigned according to the cell morphology (Bernardo et al., 2021; Gray et al., 2021). It is used to investigate the neutralizing effect of anti-toxins (Chen et al., 2020; Nguyen et al., 2020; Yang et al., 2014). The visual scoring of cell morphology is an inexpensive but laborious method (Bernardo et al., 2021; Gray et al., 2021; Nguyen et al., 2020). As with any subjective visual assessment, the score is affected by the operator and susceptible to a variety of biases. In addition, it is typically performed as an endpoint assay (Barthold et al., 2022; Bernardo et al., 2021; Chen et al., 2020; Diemert et al., 2012; Hussack et al., 2011; Yang et al., 2014). The microscope images are commonly collected and scored 24 h after incubation with the drugs (Chen et al., 2020; Yang et al., 2014). Therefore, the development of the toxic effect on cell growth over time cannot be followed unless a frequent and laborious visual inspection setup is performed (Diemert et al., 2012; Single et al., 2015). Despite these limitations, visual evaluation is still widely utilized in cytotoxicity assays (Barthold et al., 2022; Chen et al., 2020; Nguyen et al., 2020; Olling et al., 2011; Yang et al., 2014).

To address the described technical limitations, new technologies such as cell-based impedance with real-time cell analysis (CBI-RTCA) can be used (Bernardo et al., 2021). Interestingly, CBI-RTCA systems such as the CBI-RTCA eSight enable simultaneous cell imaging and impedance readouts (Li et al., 2024). The CBI-RTCA eSight system is a combination of a CBI-RTCA analysis that can perform real-time monitoring of electrical impedance with an automatic microscope (Khan et al., 2025). The system utilizes custom microplates (E-plates) whose bottom is covered by gold electrodes; thus an electric current can pass through the fluid in the wells (Bernardo et al., 2021; Kustermann et al., 2013; Limame et al., 2012). Based on the extent of cell adhesion and proliferation, the degree of freedom of the electrons’ flow varies (Bernardo et al., 2021; Kustermann et al., 2013; Rees and Thomas, 2015). This phenomenon is called impedance and the CBI-RTCA system converts it into Cell Index (CI) measurements (Bernardo et al., 2021; Qiu et al., 2016; Rees and Thomas, 2015; Stoddart, 2011). The ability of CBI-RTCA to detect changes in cell adhesion makes it a promising technology in cytotoxicity cell-based assays (Kustermann et al., 2013; Stoddart, 2011). However, it has not been widely used for neutralization studies where visual scoring is the practice in the field (Hussack et al., 2011; Qiu et al., 2016; Yang et al., 2014).

In the present study, we established and optimized a CBI-RTCA eSight workflow for toxin mediated cytotoxicity cell-based assays. We evaluated CBI-RTCA eSight platform for *C. difficile* toxin and neutralization studies and compared it to visual inspection of cell morphology. Specifically, we utilized *C. difficile* TcdA and the anti-TcdA antibody fragment, named AH3 (Yang et al., 2014). As a cell model, we used Vero cells because they are adherent cells whose prolonged shape converts into a round morphology when in contact with TcdA (Barthold et al., 2022). Vero cells have been historically utilized as cell models for *C. difficile* cytotoxicity assays (Ok et al., 2023). The results demonstrate that the CBI-RTCA analyzer is highly sensitive in detecting small cell adhesion changes at low toxin concentration and early after toxin treatment. Moreover, it is a robust technology for *C. difficile in vitro* toxin assays as it can quantify cytotoxic effect and/or toxin neutralization using drugs such as antibodies (Qiu et al., 2016). CBI-RTCA eSight further benefits from the advantage to perform both kinetic and endpoint assays together with microscopy monitoring with no or minimum workload due to its automated technology (Bernardo et al., 2021; Li et al., 2024). These features make it suitable for high throughput drug screening and *C. difficile* infections diagnosis upon toxin quantification from patients’ samples (Bernardo et al., 2021; Huang et al., 2014; Qiu et al., 2016).

## 2 Materials and methods

### 2.1 Bacterial strains, mammalian cell lines, and growth conditions

Competent *Escherichia coli* (*E. coli*) DH5α (NEB, #C2987H) were grown in Luria Bertani (LB) (Invitrogen, #11558866) medium and selective LB agar plates at 37 °C supplemented with carbenicillin (VWR International A/S, #A1491.0010) at a concentration of 100 μg/mL. African green monkey Vero-B4 cells (DSMZ, ACC33) were grown in RPMI (Gibco, #42401018) medium supplemented with 10% fetal bovine serum (Gibco, #10500064), 1% L-glutamine (Sigma-Aldrich, #G7513-100ML), and 1% penicillin-streptomycin (Fisher Scientific, #15-140-122).

### 2.2 Monitoring vero cell growth in real-time

Real-time cell behavior was monitored using the xCELLigence RTCA eSight technology (Agilent), enabling simultaneous impedance-based measurements and live-cell imaging of the same E-plate. The CBI-RTCA eSight analyzer can hold three cradles for impedance and microscopy monitoring and two for imaging only. The cradles are kept in a Heracell-240 Incubator set at 37 °C with 5% CO_2_. The device is connected to a computer recording the impedance measurements and transforming into CI readouts. Before cell seeding, 50 μL RPMI media was added to 96-well E-plates to record the background impedance. After the Resistor Plate and background check, Vero cells were seeded. A volume of 100 μL of Vero cells were dispensed at different cell densities in duplicates (from 5 × 10^3^ cells/well to 3 × 10^4^ cells/well) and settled for 30 min under sterile conditions to decrease the plate edge effect. Moreover, wells containing RPMI without cells served as a control. The growth was monitored using the CBI-RTCA eSight for ∼40 h at 37 °C and 5% CO_2_. Cell Index (CI) measurements were recorded every hour.

### 2.3 *Clostridioides difficile* TcdA cytotoxicity effect and toxin quantification

**FIGURE 1:**
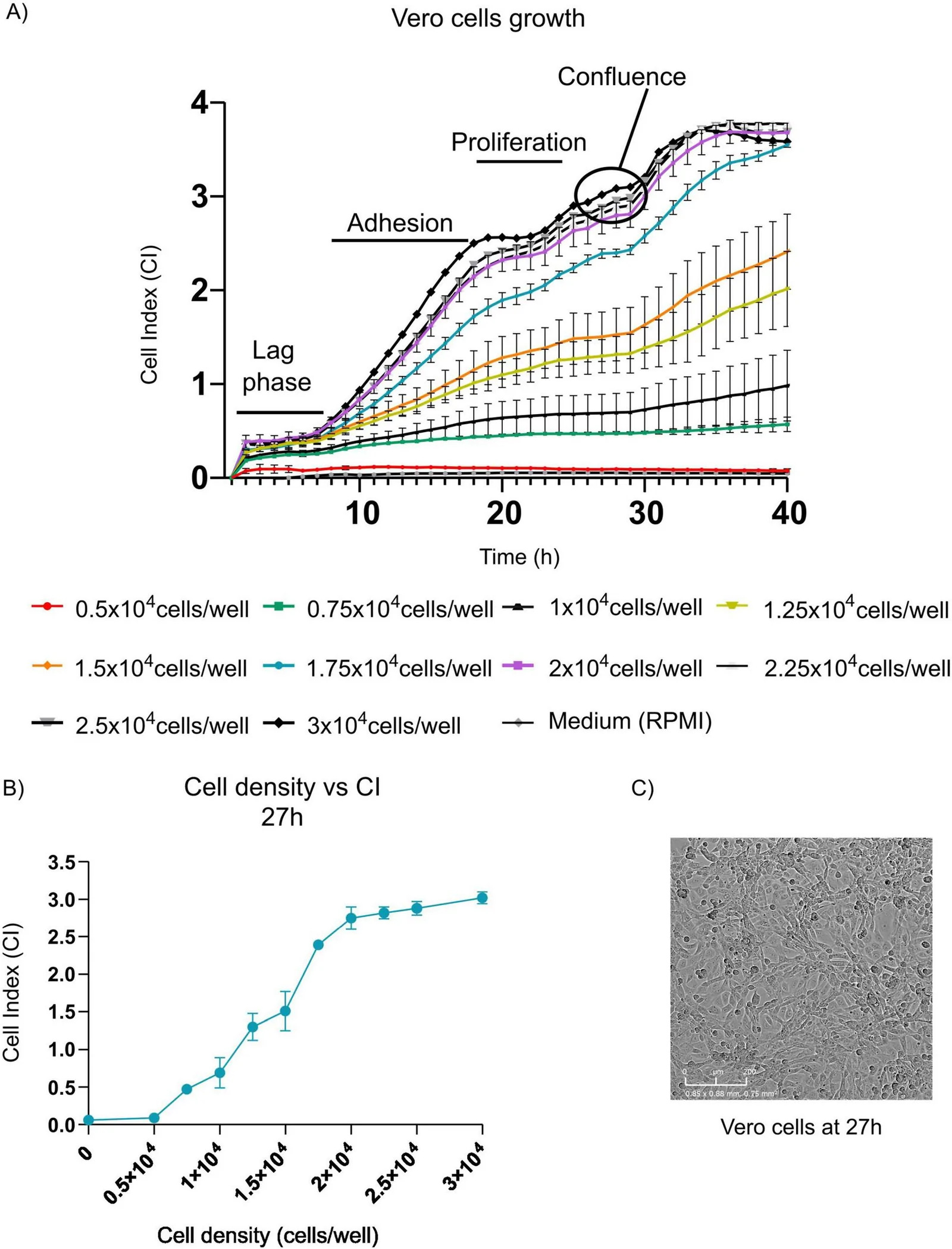
Characterization of Vero cell growth. **(A)** Cell growth characterization. Cell Index (CI) as a function of the time in hours (h). Cells reached confluency at the second plateau phase, around 25–30 h after seeding. Results from one experiment conducted in technical duplicates; **(B)** curve reporting the CI values as a function of the seeding cell density (cells/well) at 27 h after TcdA treatment; **(C)** representative microscopy image of a confluent Vero cell monolayer (3 × 10^4^ cells/well seeding cell density) at 27 h from cell seeding. Data represent mean ± standard deviation.

**FIGURE 2:**
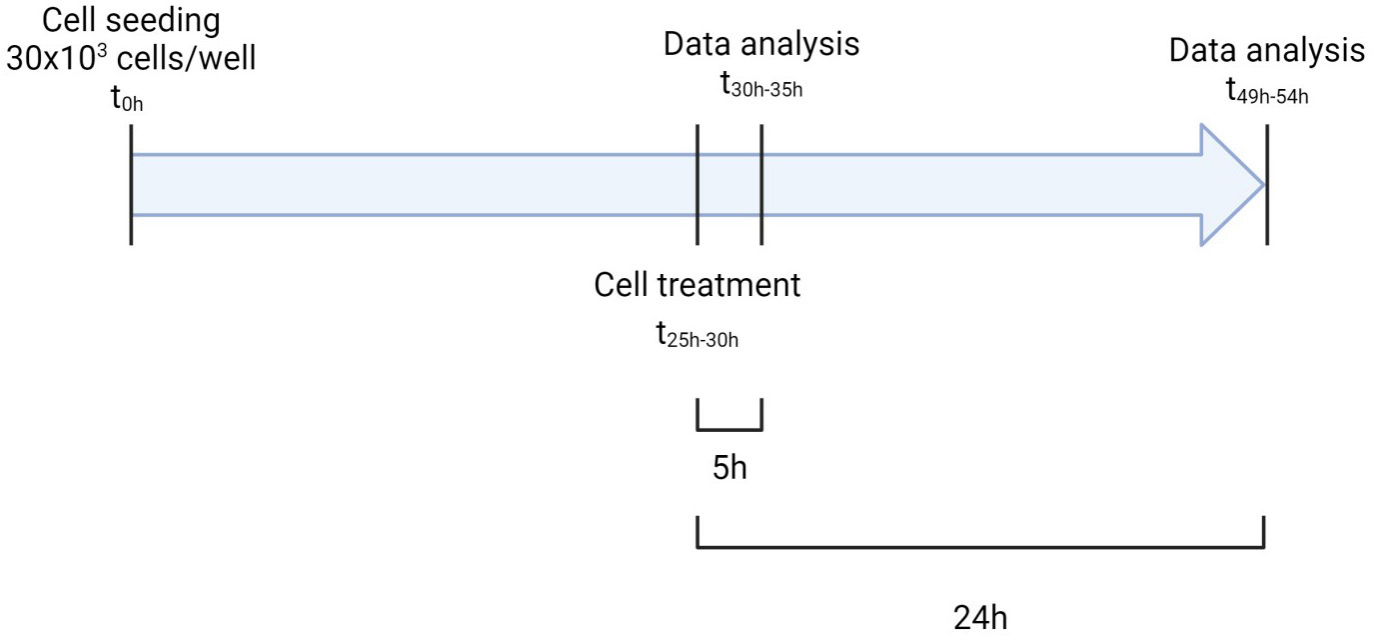
Experimental design for TcdA cytotoxicity and neutralization assay. Vero cells were seeded at a seeding cell density of 3 × 10^4^ cells/well at the time 0 h (t0h). The cells were grown for 25–30 h before reaching confluency. At 25–30 h (t25h–30h) the cells were treated. The TcdA toxic effect was monitored over 24 h. The data (CI and microscope images) for assessing TcdA cytotoxic effect were collected 5 h after treatment (t30h-35h) and 24 h after treatment (t49h–54h). Furthermore, the AH3_Fc neutralization effect was monitored over 24 h after treatment. The CI measurements and the microscopy images were collected at t49h–54 h. Figure created in BioRender.com.

To conduct the cytotoxicity assay with *C. difficile* native TcdA (Abcam, #ab124000), Vero cells were treated when they reached confluency. A cell density of 3 × 10^4^ cells/well was found to be ideal for reaching confluency in 25–30 h after seeding (Figure 1). Six dilutions of TcdA from 91 pM to 0.03 pM as well as a 0 pM control were prepared in a U-bottom pre-treatment plate. Before treating Vero cells, the pre-treatment plate was pre-warmed at 37 °C for 10 min. 50 μL of TcdA titration was added to the 96-well E-plate where Vero cells reached a confluent state. The effect of TcdA on the cell monolayer was monitored by recording the CI every 10 min. The CI data were collected at 5 and 24 h after cell treatment and analyzed by RTCA eSight Software v.1.2.1. The CI values were normalized by dividing each CI value by the CI measured 10 min after TcdA treatment. Consequently, following normalization, all wells had a normalized CI value of 1. (Valdés et al., 2015). From the normalized CI data, the values corresponding to the Area Under the Curve (AUC) and the endpoint Y values were utilized for calculating TcdA dose-response curves. See Figure 2 for the experimental design.

The TcdA dose-response curve obtained from AUC data was utilized as a model for generating a toxin quantification standard curve. The TcdA concentration giving 10% of viable cells was extrapolated from the quantification standard curve, providing a method for toxin quantification. To prove the quantification approach, 3 × 10^4^ cells/well were seeded and grew until they reached confluency. When cells were confluent, they were treated with the TcdA concentration giving 10% cell viability, obtained from the standard curve. The data were analyzed as above described.

### 2.4 TcdA neutralization assay

The neutralization assay was conducted utilizing a fixed TcdA concentration giving a 90% cytotoxicity (Qiu et al., 2016). A cell density of 3 × 10^4^ cells/well was seeded as previously described and grew until reached confluency. Five dilutions of AH3_Fc from 8.75 nM to 3.42 × 10^–6^ nM as well as with no antibody control were prepared in the pre-treatment plate and incubated with 12.5 pM TcdA. The antibody-TcdA solution was incubated for 50 min at RT under sterile conditions and at 37 °C for 10 min for a total of 1 h. Moreover, Vero cells incubated with RPMI and without either TcdA or AH3_Fc were used as Untreated control. After 1 h of incubation, 50 μL of solution was added to the 96-well E-plate with confluent Vero cells. TcdA neutralization was recorded by CI measurements every 10 min and the data were collected 24 h post-treatment. The data normalization for CI measurements (Valdés et al., 2015) was conducted as described above. Refer to Figure 2 for the experimental design.

### 2.5 Visual inspection of cell morphology

The effect of TcdA and AH3_Fc on cell morphology was determined microscopically. One brightfield microscopy image per well was captured with a 10× objective every hour to monitor cell morphological changes. The data were collected at 5 and 24 h after cell treatment with TcdA, and after 24 h after treatment with AH3_Fc. Each TcdA cytotoxicity experiment was conducted in technical duplicates, obtaining two microscopy images for each TcdA concentration as well as for the untreated control. Similarly, each neutralization experiment was performed in technical duplicates, producing two microscopy images for each AH3_Fc concentration. In addition, two microscopy images were acquired for samples treated with a fixed TcdA concentration and 0 nM AH3_Fc, as well as for the untreated control sample. The microscopy images were scored by two independent operators. The operators were trained to recognize a viable and adherent Vero cell monolayer, but also round and detached cells when treated with the high TcdA concentration. The images were scored as 0 (0% viable cells), 1 (33% viable cells), 2 (66% viable cells), or 3 (100% viable cells). The operators’ scores were averaged and utilized for generating TcdA and AH3_Fc’s dose-response curves. See Figure 2 for the experimental design.

### 2.6 Data analysis

TcdA dose-response curves at 24 h and 5 h after treatment as well as the concentration of toxin exhibiting a 50% cytotoxic effect (IC_50_) (Pan et al., 2013) were obtained utilizing GraphPad v.10 nonlinear regression fit after log_10_ transformed concentrations. “Log (antagonist) vs. response – variable slope” was chosen as a model: (Y = Bottom + (Top-Bottom)/ (1+10^ ((LogIC_50_-X) *Hill Slope))).

The AH3_Fc’s dose-response curves, the concentration exhibiting 50% response (EC_50_) (Hussack et al., 2023; Qiu et al., 2016) and the maximum cell recovery values were obtained utilizing GraphPad v.10 nonlinear regression fit after log_10_ transformed concentrations. “Log (agonist) vs. response – variable slope” was chosen as a model: (Y = Bottom + (Top-Bottom)/ (1+10^ ((LogEC_50_-X) *Hill Slope))). Statistical analysis was performed using GraphPad v.10. The statistics among two groups were performed using Student’s *t-test* while the analysis between more than two groups was conducted using One-way ANOVA. The homogeneity across standard deviations was determined using the Brown-Forsythe test, obtained from One-way ANOVA analysis (*P* < 0.05 was evaluated as significant).

## 3 Results

### 3.1 Determination of optimal seeding cell density and confluency

Before conducting the TcdA cytotoxicity and neutralization assays using Vero cells, the seeding cell density and experimental parameters had to be optimized. Vero cell growth was characterized using cell-based impedance assay with real-time cell analysis (CBI-RTCA eSight) which can measure the differences in cell proliferation and perform simultaneous microscopy (Khan et al., 2025). The change in cell adhesion and proliferation determines a change in the current passing the golden electrodes at the bottom of E-plates. The CBI-RTCA detects such changes in real time and converts them in Cell Index (CI) readouts as a function of the time (Bernardo et al., 2021; Kustermann et al., 2013). When the cells adhere and proliferate the CI increases over time; when the cells are absent or not growing the CI readouts are low (Kho et al., 2015; Oktem et al., 2016).

Ten different seeding cell densities, from 0.5 × 10^4^ cells/well to 3 × 10^4^ cells/well, were assessed to identify the optimal conditions. Multiple wells filled only with RPMI medium served as controls. The behavior of cell growth depended on the seeding cell density. The seeding cell densities ranging from 1.75 × 10^4^ to 3 × 10^4^ cells/well exhibited a 5-h lag phase, followed by a sharp increase in the CI, which indicated cell adhesion. Two phases of proliferation followed the lag phase, and they were characterized by a progressive rise in the CI. The growth phases described above were less pronounced at seeding cell densities of 1.25 × 10^4^ and 1.5 × 10^4^ cells/well, while at lower seeding cell densities (0.5 × 10^4^ to 1 × 10^4^ cells/well), the proliferation phases were either absent or flat (Figure 1A). Depending on the number of seeded cells the proliferation profile changes, except for 0.5 × 10^4^ seeded cells/well which did not present any growth. The titration of different seeding cell densities identified the time point for cells to achieve confluency. Confluency was reached between 25 and 30 h for the four highest seeded cell densities (Figure 1A). The relationship between CI and cell density at 27 h showed linearity from 0.75 × 10^4^ to 1.75 × 10^4^ seeded cells/well, while it plateaued between 2 × 10^4^ and 3 × 10^4^ seeded cells/well reflecting the confluency status (Figure 1B). The four highest cell densities reached a CI ∼3 (Figure 1A) demonstrating a weak but suitable adhesion (Kho et al., 2015). A representative image of a cell monolayer at 27 h after seeding is shown in Figure 1C.

### 3.2 The CBI-RTCA system measures the effect of TcdA on vero cells and can be utilized for toxin quantification

**FIGURE 3:**
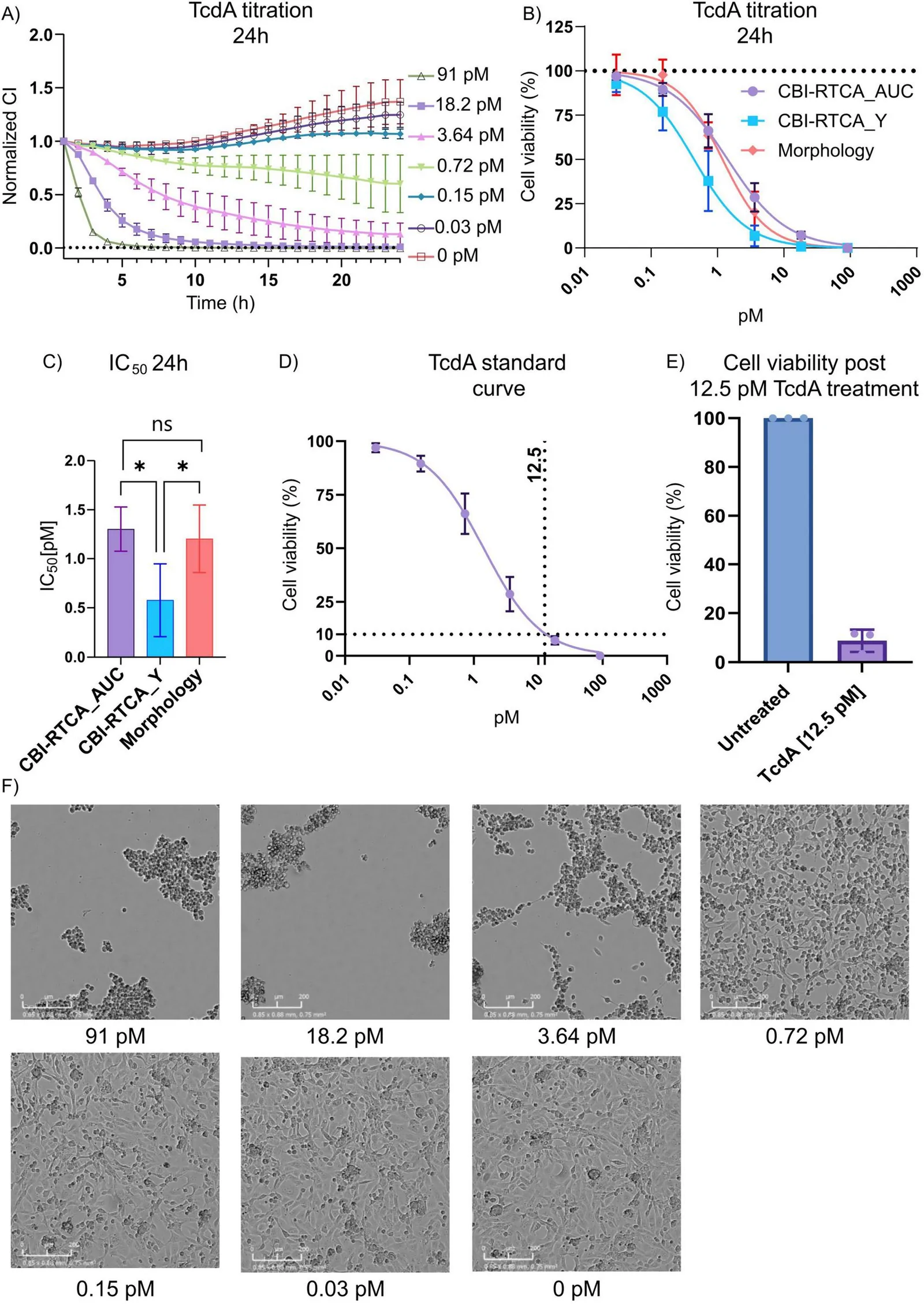
TcdA cytotoxic effect on Vero cells at 24 h post-treatment. **(A)** TcdA effect on Vero cells treated with different toxin concentrations. Control wells were incubated with RPMI medium only (0 pM); **(B)** the image shows two dose-response curves from data collected by CBI-RTCA considering the Area Under the Curve values (CBI-RTCA_AUC) and the endpoint measurements (CBI-RTCA_Y). The third dose-response curve was calculated via visual scoring (Morphology). The dose-response curves were obtained by plotting the cell viability in percentage (%) as a function of TcdA concentration [pM]; **(C)** comparison of IC50 (TcdA concentration required to reduce the 50% cell response) values obtained with the three different data analysis approaches; **(D)** TcdA standard curved obtained from CBI-RTCA_AUC data by plotting the cell viability in % as a function of TcdA concentration [pM]; **(E)** effect of 12.5 pM TcdA on cell viability in %; **(F)** representative brightfield microscopy images of the effect of TcdA on Vero cell morphology. The images were utilized for morphology visual scoring. Data in (**A–D)** show the mean ± standard deviation. ns = non-significant, *P < 0.05. *n* = 4. Data in (**E**) show the mean ± standard deviation, *n* = 3.

Vero cells are commonly used as cytotoxicity models for *C. difficile* toxins (Ok et al., 2023). They have a prolonged shape and adhere to culture flasks (Nguyen et al., 2020), but upon exposure to the toxins they round up and detach (Barthold et al., 2022; Hamo et al., 2021). The CBI-RTCA system detects these changes and converts them into CI measurements, which are subjected to a drop (Bernardo et al., 2021; Huang et al., 2014). To investigate the relationship between the effects of TcdA and the CI measurements, a titration of the toxin was incubated with a confluent Vero cell monolayer keeping an untreated sample (0 pM TcdA) as control. Based on the above optimization studies on seeding cell density and time of confluency, we utilized a seeding cell density of 3 × 10^4^ cells/well and grew it for 29 h, when it reached confluency. Confluent Vero cells were treated and monitored for 24 h. The TcdA titration results show that the increase in TcdA concentrations resulted in a corresponding decrease in CI values, demonstrating an inverse correlation. At the lowest TcdA concentration, the CI curve remained close to the control, while the highest concentration (91 pM) produced the lowest CI values. Notably, at 5 h, the CI for the highest TcdA concentration had already dropped almost to zero (Figure 3A).

The titration of the six TcdA concentrations ranging from 0.03 pM to 91 pM in four independent experiments and technical duplicates was utilized to evaluate the TcdA concentration limit. A significant (*P* < 0.05) reduction in CI was detected at 0.15 pM TcdA concentration.

To validate the applicability and potential advantages of the CBI-RTCA analyzer in assessing the cytotoxic effects of *C. difficile* TcdA, we compared its performance with traditional cell morphology scoring. To this end, we compared CBI-RTCA endpoint readouts at 24 h (Y values), with the visual inspection results. Unlike endpoint assays, CBI-RTCA monitors cellular changes in real-time, capturing the kinetics of cell growth (Diemert et al., 2012; Kho et al., 2015; Pan et al., 2013). The Area Under the Curve (AUC) analysis provides data on these kinetic changes (Pan et al., 2013), and we compared AUC with Y values and visual inspection outcomes. To evaluate the toxic effects of TcdA on Vero cells, three dose-response curves were generated from TcdA titration (Figure 3B). Two curves were based on CBI-RTCA data: one from AUC and the other from endpoint Y values. The third curve was derived from the visual inspection of cell morphology. The CBI-RTCA dose-response curve, when displayed as an endpoint assay, showed a higher potency than the AUC and morphology curves. In contrast, the AUC and morphology curves presented the same potency (Figure 3B). The differences in potency were reflected by the IC_50_ values: the IC_50_ of 1.3 pM TcdA from the CBI-RTCA AUC curve was 2-fold significantly higher than the Y curve IC_50_ of 0.58 pM. The TcdA potency calculated from morphology scoring was significantly lower (IC_50_ = 1.20 pM) than that obtained from CBI-RTCA endpoint measurements, and comparable to the AUC-based assessment (Figure 3C).

To investigate the applicability of CBI-RTCA for toxin quantification, we utilized the CBI-RTCA_AUC curve as a model for building a standard curve. The standard curve spanned from 100% to 0% of viable cells when they were untreated and treated with 91 pM TcdA, respectively (Figure 3D). To demonstrate the potential of CBI-RTCA as a quantification method, we showed that we can return to a toxin concentration given a certain cell viability response. From the standard curve we identified, on the Y-axis, the 10% of viable cells and calculated back the toxin concentration, resulting in 12.5 pM (Figure 3D). 12.5 pM TcdA was used to treat 3 × 10^4^ cells/well in a confluent state. The accuracy of the quantification assay is confirmed by Figure 3E. The bar plot shows that the cells reached ∼10% viability when treated with 12.5 pM TcdA (Figure 3E).

Morphological changes induced by TcdA are shown in Figure 3F where healthy Vero cells (0 pM TcdA) displayed an elongated shape (Nguyen et al., 2020). After TcdA exposure, cells rounded, detached, and formed clumps as the toxin concentration increased. The relationship between TcdA and morphology modifications is concentration-dependent, supporting the previous CBI-RTCA readouts (Figure 3A).

### 3.3 CBI-RTCA system is more sensitive than visual inspection

**FIGURE 4:**
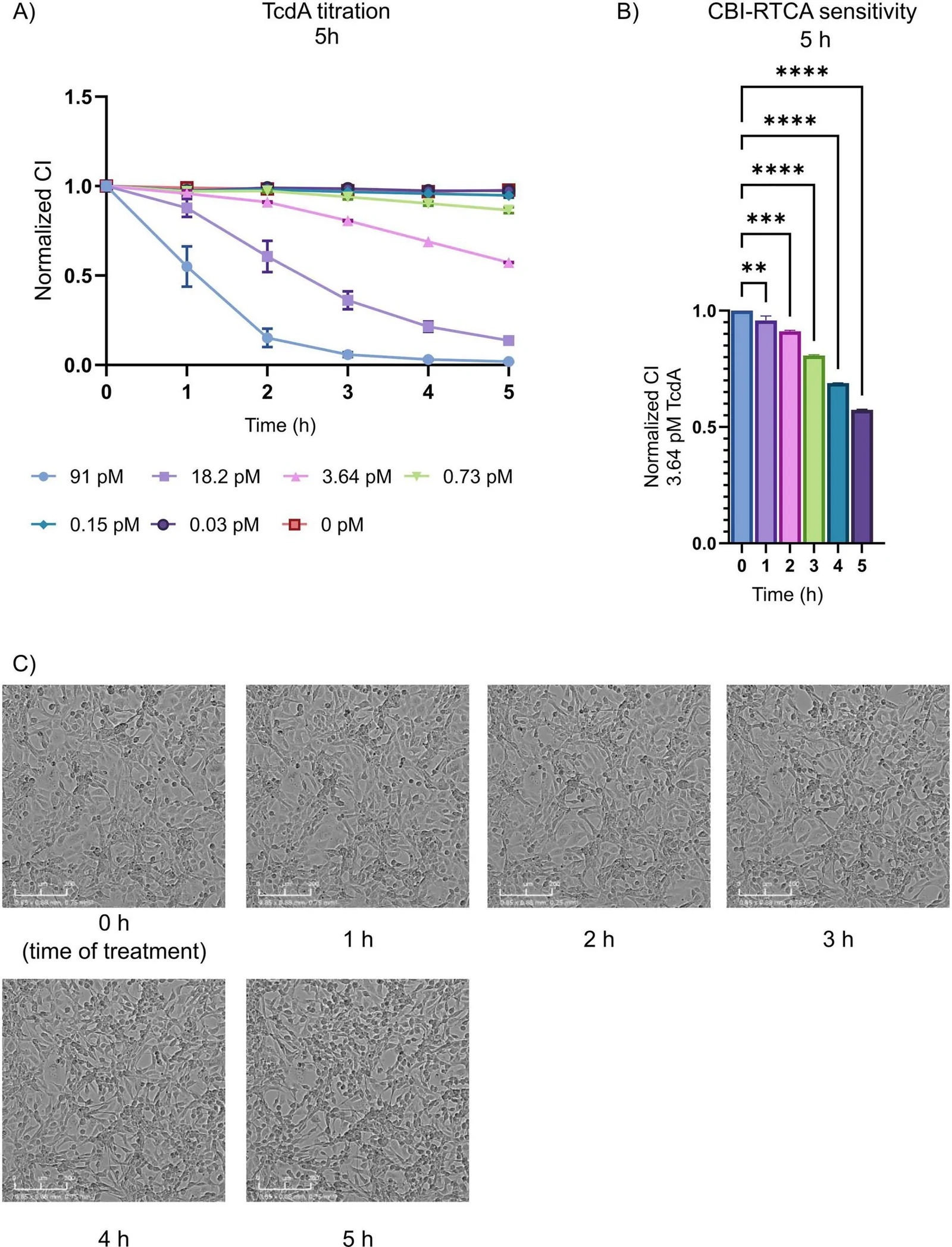
TcdA monitoring within 5 h from treatment of Vero cells. **(A)** CBI-RTCA normalized Cell Index (CI) readouts of TcdA titration in [pM] as a function of the time in hours (h), *n* = 2; **(B)** normalized CI over 5 h post-treatment at 3.64 pM TcdA. The data report the following SDs: ± 0.018 for 1 h, ± 0.004 for 2 h, ± 0.003 for 3 h, ± 0.0005 for 4 h, ± 0.002 for 5 h. The experiment was performed in technical duplicates and three biological replicates. Data represent the mean ± standard deviation. ns = non-significant, **P < 0.01, ***P < 0.001, ****P < 0.0001. Data were analyzed via One-way ANOVA; **(C)** representative brightfield microscopy images of TcdA at a concentration of 3.64 pM at different time points.

To further investigate the discrepancy between CBI-RTCA measurements and visual inspection, we conducted an additional assessment at 5 h post-treatment. This time point was selected because the highest concentration of TcdA (91 pM) had reached its maximum effect (Figure 4A). To track the changes and compare CBI-RTCA CI with visual inspection readouts, we selected the TcdA concentration of 3.64 pM. This concentration provided an optimal comparison between the two methodologies, as it avoided immediate effects observed at high concentrations and delayed effects at low concentrations. At 3.64 pM, we were able to observe more discernible morphological changes over time. The CBI-RTCA system monitored the response to 3.64 pM TcdA, detecting significant changes already after 1 h (*P* < 0.05). The reduction in CI over time was more pronounced between hours 3 and 5 (*P* < 0.0001) (Figures 4A,B). By the 5-h mark, the CBI-RTCA system recorded a clear reduction in CI, whereas morphological changes observed via visual inspection were minimal, becoming visible when the CI decreased by more than 0.25 points at hour 5 (Figures 4A,C).

**FIGURE 5:**
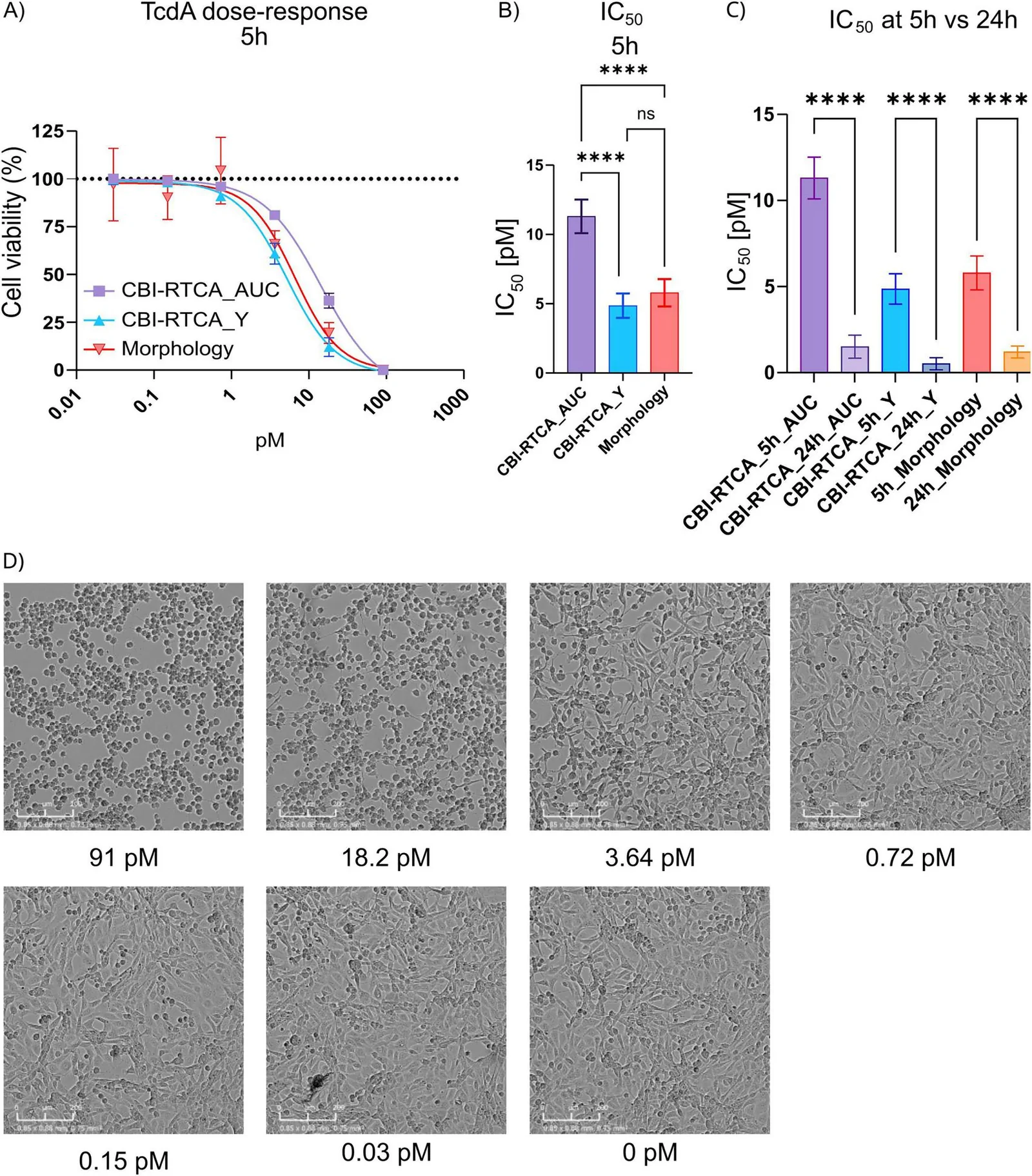
TcdA effect 5 h after treatment of Vero cells. **(A)** Dose-response curves from data collected by CBI-RTCA Area Under the Curve (CBI-RTCA_AUC) and endpoint (CBI-RTCA_Y) data. The third dose-response curve was generated after visual scoring (Morphology). The dose-response curves were generated by plotting the cell viability in percentage (%) as a function of TcdA concentration [pM] reported as anti-log of log-transformed values, *n* = 4; **(B)** comparison of IC50 (TcdA concentration to diminish the 50% the cell response) values obtained with the three different data analysis methods; **(C)** comparison of IC50s at 5h vs. 24h within the same methods; **(D)** representative brightfield microscopy images of the effect of TcdA on Vero cell morphology. The images were utilized for morphology visual scoring; Data represent the mean ± standard deviation. ns = non-significant, ****P < 0.0001. *n* = 4.

CBI-RTCA readouts (AUC and Y values) were compared to the visual assessment of Vero cell morphology 5 h post-treatment, generating three dose-response curves from the collected data. The TcdA effect measured via AUC (IC_50_ = 11.30 pM) showed 2-fold lower potency than that derived from CBI-RTCA Y values (IC_50_ = 4.86 pM) and visual inspection (IC_50_ = 5.79 pM) (Figures 5A,B).

Comparing TcdA effects at 5 and 24 h, the IC_50_ TcdA potency measured by CBI-RTCA was 10-fold higher at 24 h than at 5 h when determined by AUC values, and similarly 8-fold higher from CBI-RTCA Y readouts. The same pattern was identified for visual inspection with a lower potency at 5 h than at 24 (Figure 5C). The results suggest an increase in TcdA potency with longer incubation, as indicated by the decreased IC_50_ values. Representative brightfield microscopy images of the TcdA effect on Vero cell morphology are reported in Figure 5D. An example of healthy cells is depicted in the 0 pM sample. On the other hand, the cells showed an increased degree of cell rounding as a function of TcdA concentration.

### 3.4 Determination of drug neutralization capacity of a sdAb-Fc fusion antibody against TcdA using CBI-RTCA

**FIGURE 6:**
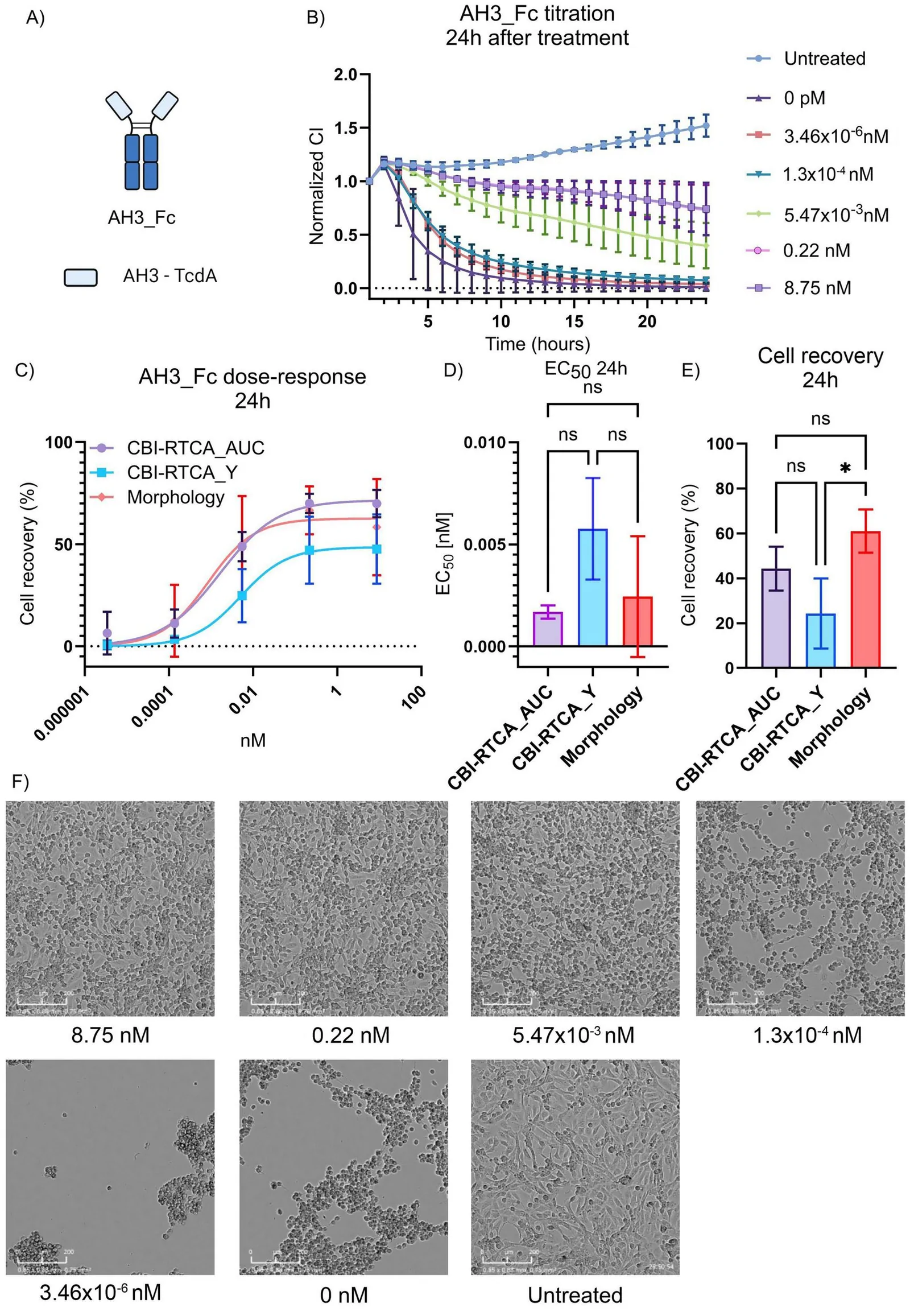
AH3_Fc effect at 24 h after Vero cells’ treatment. **(A)** Schematic representation of the single-domain antibody Fc-fusion construct AH3_Fc; **(B)** AH3_Fc titration effect on Vero cells. Cells were treated with 12.5 pM fixed toxin concentration and AH3_Fc titration starting from 8.75 nM to 0 nM. The 0 nM sample corresponds to cells treated with 12.5 pM TcdA and 0 nM AH3_Fc. The Untreated sample indicates cells that are not treated with any substance. Control wells were incubated with RPMI medium only; **(C)** dose-response curves of AH3_Fc from data collected by CBI-RTCA from the Area Under the Curve measurements (CBI-RTCA_AUC) and the endpoint values (CBI-RTCA_Y). The third dose-response curve was generated after the visual inspection of Vero cell morphology. Y-axis = cell recovery in percentage (%), X-axis = concentration of AH3_Fc [nM] reported in anti-log after log-transformation of the concentrations; **(D)** EC50 [nM] (antibody concentration giving 50% of the response) comparison; **(E)** maximum cell recovery comparison reported in %; **(F)** representative images of AH3_Fc neutralization effect on Vero cells after TcdA treatment utilized for visual scoring. Data represent the mean ± standard deviation. Experiments were conducted in technical duplicates and three independent biological replicates; ns = non-significant, *P < 0.05.

CBI-RTCA monitors changes in CI (Bernardo et al., 2021; Pauly et al., 2012) and to verify its ability to quantify TcdA neutralization, we validated and conducted a neutralization assay. We utilized a model anti-TcdA antibody, generated by genetically fusing the single domain antibody (sdAb) AH3 (Yang et al., 2014) to the hinge region of a Fc IgG1. The resulting molecule, named AH3_Fc has 2 sdAbs as 2 TcdA binding sites (Figure 6A). AH3 has been described to neutralize TcdA by blocking its pH-dependent conformational change after toxin internalization, thereby preventing the activation of the intracellular signaling pathway which leads to cell rounding (Chen et al., 2022; Kordus et al., 2022). For the *in vitro* neutralization assay, TcdA [12.5 pM] was added to confluent cells with various concentrations of AH3_Fc. CI measurements were monitored by RTCA over the time course of 24 h. We used 24 h CBI-RTCA monitoring to enable comparison with visual assessment of cell morphology, which is commonly performed at this time point in previous studies (Chen et al., 2020; Yang et al., 2014). The 24 h time point also enabled comparison with previously published data, supporting CBI-RTCA validation.

The untreated control exhibited the highest CI values, while the addition of 12.5 pM TcdA without the neutralizing sdAb Fc-fusion construct (0 nM) resulted in the lowest CI. The addition of AH3_Fc restored the CI in a concentration-dependent manner. At concentrations of 8.75 nM and 0.22 nM, AH3_Fc showed the highest and comparable neutralization, indicating that a saturation has been achieved. However, AH3_Fc did not reach full TcdA neutralization (Figure 6B).

*In vitro* neutralization assays using antibody-based drugs are often evaluated by visual scoring of morphological changes (Chen et al., 2020; Yang et al., 2014). To explore the utility of the CBI-RTCA analyzer in the neutralization assays, we followed the same experimental workflow used for TcdA. Thus, we compared the three evaluation methods: CBI-RTCA AUC, CBI-RTCA endpoint Y values, and visual scoring. CBI-RTCA data for AUC and Y values as well as brightfield microscopy images were collected 24 h post-treatment. The images were compared to cells treated only with 12.5 pM TcdA (0 nM AH3_Fc) and healthy untreated control (no TcdA and AH3_Fc) (Figure 6F). We generated three dose-response curves using CBI-RTCA AUC, Y values, and manual morphology evaluation. AH3_Fc’s potency derived from CBI-RTCA Y data was not significantly different from that obtained from AUC measurements or morphology evaluations (Figures 6C,D). However, CBI-RTCA_Y measurements and visual scoring exhibited the greatest and comparable variability, and this might have affected the statistical analysis with AUC values. Moreover, CBI-RTCA AUC is the most reproducible system as its variability is significantly lower (*P* < 0.0001) than the two endpoint assays.

We assessed the neutralizing effect of AH3_Fc based on its ability to achieve complete cell recovery. None of the three evaluation methods showed complete recovery with AH3_Fc. Instead, cell recovery ranged between 20% and 60% across all methods. Interestingly, the highest recovery was observed when assessed by morphological evaluation and was significantly different from the CBI-RTCA endpoint data. In contrast, no significant differences were detected when compared with the AUC results (Figures 6C,E).

The neutralizing effect of AH3_Fc in promoting Vero cell recovery after TcdA treatment is illustrated in Figure 6F. Cells were treated with a fixed concentration of TcdA (12.5 pM) and a titration of AH3_Fc. As the antibody concentration decreased, the cells became more rounded and detached from the culture plate. Consistent with CI curves (Figure 6B), a concentration-dependent correlation is reported between AH3_Fc and morphology changes. Moreover, the incomplete cell recovery is further confirmed by microscopy. The highest concentration of AH3_Fc (8.75 nM) did not prevent cells from rounding when compared to the Untreated control.

## 4 Discussion

CBI-RTCA biosensor technology can measure impedance changes according to modifications in proliferation, morphology, adhesion, and invasion of cells seeded on the E-plates (Diemert et al., 2012; Lago-Baameiro et al., 2023; Li et al., 2023; Rakers et al., 2014). The morphological changes can be induced by treating cells with toxins (Gray et al., 2021; Hamo et al., 2021; Yang et al., 2014). The role of *C. difficile* TcdA in altering cell morphology was studied already in 1990 when Fiorentini et al. looked at the induction of cell rounding upon TcdA treatment (Fiorentini et al., 1990). Similarly, also TcdB induces cell rounding (Barthold et al., 2022; Davies et al., 2013; Ok et al., 2023; Valdés et al., 2015). The effect of such toxins as well as their neutralizers, is usually assessed by scoring morphological changes using microscopy (Chen et al., 2020; Olling et al., 2011; Yang et al., 2014). However, this approach is limited to the operator’s judgment, and it results in a semi-quantitative assay (Gray et al., 2021). As a response, we systematically validated and optimized the use of CBI-RTCA eSight in the present study as a technology for cell-based cytotoxicity assays. Particularly, we utilized TcdA and an anti-TcdA antibody to compare CBI-RTCA with visual scoring readouts. We established CBI-RTCA as an advantageous technology for drug screening for *C. difficile* toxin neutralization.

The first step in establishing a robust quantitative assay for *C. difficile* TcdA using CBI-RTCA was to determine when Vero cells, utilized in the present study as a cell model, reached confluency. Therefore, we characterized Vero cells’ proliferation profile using impedance measurements. The growth of Vero cells lines up with what is reported in the literature (Pauly et al., 2012). Differences in the time frames when the proliferation steps happen is related to the use of different media compositions (Slivac et al., 2010) which impact cell growth. For conducting our study, we identified a range of time between 25 and 30 h when the optimized seeding cell density of 3 × 10^4^ cells/well reached confluency (Figure 1). Once the growth conditions were optimized, the effect of TcdA was characterized by CBI-RTCA monitoring as well as visual scoring over 24 h post-treatment. CBI-RTCA can measure changes in cell adherence via CI readouts, confirmed by microscopy morphological changes (Figures 3A,F). TcdA potency ranged into the low pM concentrations when monitored after 24 h (Figures 3B,C), lining up with previous findings (Kreimeyer et al., 2011). The TcdA IC_50_ values for BCI-RTCA_Y and morphology evaluation showed a significant difference at 24 h monitoring (Figure 3C). On the contrary, the values were not significantly different after 5 h monitoring (Figure 5B). These results suggest a time-dependent TcdA cytotoxicity, in alignment with other studies (Brito et al., 2002; Gerhard et al., 2005; Jochim et al., 2011; Keel and Songer, 2011). Importantly, the ability of CBI-RTCA to continuously monitor changes in cell adhesion allows these time-dependent effects to be captured in real time (Khan et al., 2025; Pan et al., 2013), providing additional information on toxin-induced cellular responses compared with endpoint morphological assessment.

To characterize CBI-RTCA utility in drug screening, we focused on TcdA effect at 3.64 pM at 5 h post-treatment. Before this time point, no visible signs of TcdA cytotoxicity were detectable by eye, rather only the CBI-RTCA can measure CI changes before 5 h. CBI-RTCA detected significant changes in cell adherence already at 1 h post-treatment, while it reduced the measurements of 0.31 and 0.43 point at 4 and 5 h, respectively (Figure 4B). Such discrepancies between visual inspection and CBI-RTCA measurements highlight the CBI-RTCA as a high sensitivity technology, corroborating previous studies (Gray et al., 2021; Limame et al., 2012). Given CBI-RTCA sensitivity, label-free and real-time system (Gray et al., 2021; Kustermann et al., 2013), it can be exploited for diagnostic strategies, especially toxin quantification (Huang et al., 2014; Ryder et al., 2010; Shen et al., 2024). Recent studies have demonstrated the applicability and high sensitivity of CBI-RTCA for TcdB quantification in *in vitro* assays using cell lines, as well as in clinical stool samples (Huang et al., 2014; Ryder et al., 2010; Shen et al., 2024). In agreement with these findings, we confirmed and validated the suitability of CBI-RTCA for toxin quantification and further expanded its application to TcdA quantification in *in vitro* assays using Vero cells. We generated a toxin quantification standard curve, here based on the percentage of the cell viability as a function of TcdA concentration. As proof, we quantified the toxin concentration giving 10% cell viability (Figures 3D,E). Such approach guarantees a fast high sensitivity method that can be applied to determine unknown TcdA concentrations, for instance from human samples, by using the optimized assay and for other toxins such as TcdB. However, the reported quantification method must be optimized for each toxin independently as TcdA and TcdB differ for mechanism of action and cytotoxic profiles (Aktories et al., 2017; Di Bella et al., 2016).

The ability of CBI-RTCA eSight to determine the drug neutralization capacity has been explored by using the model anti-TcdA antibody AH3_Fc. AH3_Fc neutralized TcdA and, when assessing its capacity as a kinetic assay (CBI-RTCA _AUC), it showed less variability suggesting to be a high reproducible method (Figure 6D). AH3_Fc did not completely neutralize TcdA, which is seen to reflect the partial neutralizing activity of AH3_Fc rather than a limitation of assay robustness. Partial neutralization of toxins has been reported in several studies and may result from the complexity of toxin structure, molecular interactions, and mechanisms of action (Hussack et al., 2011; Hussack et al., 2018). Recent studies have shown that combining anti-TcdA sdAbs targeting distinct epitopes can enhance neutralization potency (Hussack et al., 2023). AH3 interferes with the pH-dependent conformational change of the glucosyltransferase domain, (Chen et al., 2022) suggesting that combining AH3 with an sdAb targeting a different TcdA epitope may provide complementary inhibition and improve overall neutralization.

TcdA neutralization (Figures 6D,E) and cytotoxicity results (Figures 3C, 5B) do not show consistent differences across CBI-RTCA kinetic and endpoint assays and morphology assessment, validating CBI-RTCA’s suitability and advantages for neutralization and cytotoxicity studies. Both assays can be equally used without influence on the results, but CBI-RTCA is preferred because it is faster, automated, and less dependent on the operator judgment (Bernardo et al., 2021; Witkowski et al., 2010).

A key advantage of CBI-RTCA is its ability to run rapid kinetic assays that track drug effects on cell growth over time (Bernardo et al., 2021; Pauly et al., 2012). This function allows detection of early cellular changes that may be missed by endpoint CBI-RTCA assays and by visual morphology inspection (Figure 4). Therefore, CBI-RTCA kinetic assays can be considered a more accurate and robust approach for neutralization studies.

In conclusion, the present study confirms the CBI-RTCA as a technology applicable to *in vitro C. difficile* toxin assays, and it offers the advantages of a quantitative, objective, and reproducible approach (Gray et al., 2021; Pauly et al., 2012). The CBI-RTCA biosensor analyzer provides multiple advantages over microscopy-based scoring. The possibility of running multiple plates simultaneously increases the high throughput screening of drugs, guaranteeing data deprived of subjective biases (Bernardo et al., 2021; Gray et al., 2021). Moreover, CBI-RTCA is suited for investigating the kinetics of cell growth and thus the toxic or neutralization potential of a particular substance (Kho et al., 2015; Pan et al., 2013; Valdés et al., 2015). In contrast, endpoint assays do not explain toxic effects before a determined time point (Rakers et al., 2014). Still, CBI-RTCA is affected by one main limit. The biosensor system measures cell viability but it cannot determine if the cytotoxic effect derives from a drug that induces cell over-growth (Bernardo et al., 2021). To avoid this problem, it is recommended to verify the absence of any toxic effect induced by extended cell growth, for instance using simultaneous microscopy assessment (Bernardo et al., 2021). To this aim, the CBI-RTCA eSight offers a superior benefit to other CBI-RTCA instruments. The simultaneous impedance and image recording in real-time guarantees less laborious and reliable monitoring over running the CBI-RTCA and microscopy analysis separately (Li et al., 2024).

Overall, this study proved the applicability of CBI-RTCA as a robust and sensitive approach for *C. difficile* TcdA *in vitro* assays.

## Data Availability

The raw data supporting the conclusions of this article will be made available by the authors, without undue reservation.
